# Functional coupling between auditory memory and verbal transformations

**DOI:** 10.1038/s41598-024-54013-z

**Published:** 2024-02-12

**Authors:** Hirohito M. Kondo, Ryuju Hasegawa, Takahiro Ezaki, Honami Sakata, Hao Tam Ho

**Affiliations:** 1 School of Psychology, Chukyo University, 101-2 Yagoto Honmachi, Showa, Nagoya, Aichi 466-8666 Japan; 2 Research Center for Advanced Science and Technology, The University of Tokyo, Tokyo, Japan; 3 Département d’études Cognitives, École Normale Supérieure, Paris, France

**Keywords:** Human behaviour, Sensory processing

## Abstract

The ability to parse sound mixtures into coherent auditory objects is fundamental to cognitive functions, such as speech comprehension and language acquisition. Yet, we still lack a clear understanding of how auditory objects are formed. To address this question, we studied a speech-specific case of perceptual multistability, called verbal transformations (VTs), in which a variety of verbal forms is induced by continuous repetition of a physically unchanging word. Here, we investigated the degree to which auditory memory through sensory adaptation influences VTs. Specifically, we hypothesized that when memory persistence is longer, participants are able to retain the current verbal form longer, resulting in sensory adaptation, which in turn, affects auditory perception. Participants performed VT and auditory memory tasks on different days. In the VT task, Japanese participants continuously reported their perception while listening to a Japanese word (2- or 3-mora in length) played repeatedly for 5 min. In the auditory memory task, a different sequence of three morae, e.g., /ka/, /hi/, and /su/, was presented to each ear simultaneously. After some period (0–4 s), participants were visually cued to recall one of the sequences, i.e., in the left or right ear. We found that delayed recall accuracy was negatively correlated with the number of VTs, particularly under 2-mora conditions. This suggests that memory persistence is important for formation and selection of perceptual objects.

## Introduction

Our ability to understand language, to recognize voices, or to enjoy music depends on our capacity to parse complex sound mixtures into coherent perceptual objects. Perceptual objects are shaped by bottom-up sensory processing and top-down schema matching^1^. Perceptual multistability, that is, subjective experiences dissociated from sensory inputs, provides us with clues to probe mechanisms of auditory perceptual organization. In verbal transformations (VTs), spontaneous changes of auditory objects are induced by listening to a word repeated continuously over time. For example, ‘stress’ may be transformed into a variety of verbal forms, such as ‘dress’, ‘florist’, and ‘Jewish’^2,3^.

It has been found that VTs are produced by phonetic distortions of stimuli^3–5^, regrouping of perceptual segments^6^, lexical and semantic transformations^3,7,8^, and listener’s knowledge^9,10^. Some phonemes in stimuli can be replaced by phonetically similar ones when participants listen to a repeated pseudoword^11^. VTs may also be induced by schema matching that relies on the lexical content of the repeated word. When the repeated word has emotional valence, e.g., ‘rape’, participants sometimes report rather violent verbal forms, such as ‘rake’, ‘break’, and ‘wrench’^3^. Intriguingly, the type of VT further depends on linguistic experience of listeners. Elderly people (above 60 years) tend to provide verbal forms related to their knowledge, whereas children (6–10 years) frequently report nonsense words^10^. Based on previous findings, it has been argued that satiation and criterion shift are involved in VTs^4,8,12,13^.

Sensory adaptation is a key concept in this framework. Repeated listening to a word causes adaptation to the current verbal form, which is then abruptly changed to another form by a criterion shift. Computational models have elaborated dynamics of perceptual multistability using three ingredients: adaptation, inhibition, and noise^14–17^. A recent study proposed that formation and selection of auditory objects originate from multiple hierarchical levels: peripheral-, central-, and object-analysis stages^18^. In general, auditory inputs are passively stored in memory for a few seconds to search mental lexicon and understand speech^19–21^. Acoustic features of inputs are tied to a probabilistic interpretation of auditory objects in the scene, resulting in individual differences in perceptual multistability. For VTs, auditory memory is needed for comparisons between the current verbal form and new one. However, it is still unknown whether auditory memory is associated with VTs. We hypothesized that long persistence of auditory memory leads to slow adaptation of sensory inputs, which retains current verbal forms and reduces the number of VTs.

We used auditory memory and VT tasks to look for sensory–perceptual correlations. For the VT task, auditory stimuli consisted of 2- and 3-mora words. A mora is a basic unit in Japanese and generally shorter than a syllable in English. Auditory memory would be readily adapted by stimulation of 3-mora words, because verbal forms in 3-mora words account for a larger proportion of memory capacity than those in 2-mora words. In addition, VTs are more frequent for non-resegmental words than for resegmental words, such as ‘life/fly’ and ‘lips/slip’^13^. Two- and three-mora words used in this study were resegmental and non-resegmental, respectively. This would be expected to result in the larger number of VTs in 3-mora words than in 2-mora words.

Also, we accurately measured auditory memory persistence using partial-report and full-report methods. In a partial-report method, different sets of three consecutive morae were simultaneously presented to the right and left ears. Then, following visual cues indicating right or left, participants recalled a set of the three morae heard from that ear. Partial-report accuracy a few seconds after stimulus presentation would be superior to full-report accuracy for immediate recall of all six morae^19^. Thus, delayed responses in partial reports are thought to better reflect the persistence of auditory memory. We further assumed that 3-mora veridical forms are more robust in terms of the schema template than 2-mora veridical forms, and are less susceptible to other possible forms. Therefore, we expected that 2-mora conditions, relative to 3-mora conditions, would reflect pure adaptation to verbal forms within auditory memory, leading to significant correlations between VTs and auditory memory.

## Methods

### Ethics statement

This study was approved by the Research Ethics Committee of Chukyo University (approval no. RS20-017) and carried out in accordance with Ethical Guidelines for Medical and Biological Research Involving Human Subjects. Participants gave written informed consent after procedures had been fully explained to them. They were paid for their participation.

### Participants

Forty-six college students were recruited for the experiment. All participants self-reported being right-handed with normal or corrected-to-normal vision. An audiometer was used to verify that they had normal hearing. According to an a priori power analysis, we required 46 participants to detect significant correlations: *r* = 0.40, bivariate normal model; *α*-level = 0.05; and 1 − *β* = 0.80. One participant was excluded from subsequent analyses because he/she did not show any VT effect. The remaining 45 participants (23 men and 22 women) were between 19 and 26 years of age (mean ± SD age = 21.0 ± 2.4 years).

### Stimuli and procedures

Participants performed VT and auditory memory tasks on different days. Auditory stimuli were presented diotically through Sennheiser HD 599 headphones. The volume was set at a sound pressure level of 70 dB using an artificial ear (TYPE2015, ACO, Tokyo). Stimulus presentation and response collection were managed using a PC running Presentation software (version 22.1) (Neurobehavioral Systems, Berkeley, CA, USA).

The VT task consisted of four 5-min trials. In each trial, one of the following 2- or 3-mora words was played repeatedly without gaps: ‘hana’ (nose or flower in English), ‘sushi’, ‘tokei’ (clock or watch in English), and ‘banana’. Durations of the words were: ‘hana’, 297 ms; ‘sushi’, 327 ms; ‘tokei’, 337 ms; and ‘banana’, 340 ms. All words were spoken by a female native Japanese speaker. We focused on four types of verbal forms: veridical, lexical different, pseudoword, and nonsense (or undecided) forms. Possible forms for each stimulus word were explained to participants beforehand. For example, possible forms of ‘banana’ (veridical form) were: ‘nappa’ (lexically a different form; vegetables in English), ‘namba’ (pseudoword form), and ‘na-na-’ (nonsense form). Possible forms for the other trials were ‘hana’, ‘ana’ (a hole in English), ‘narha’, and ‘ha-ha-’; ‘sushi’, ‘ushi’ (a cow in English), ‘shiisu’, and ‘shi-shi-’; ‘tokei’, ‘keito’ (yarn in English), ‘kei’, and ‘to-to-.’ These possible forms were specified in previous and preliminary studies^22^. Participants were instructed to listen to a repeated word and indicate their perception whenever VTs occurred. Their responses were collected at a sampling rate of 1 kHz via four assigned keys that represented possible verbal forms of the stimulus word. A key press indicating a response was held until a subsequent key press. Test trials were preceded by a 1-min training block, in which participants practiced reporting their perception confidently and precisely^23,24^. The order of test trials was randomized across participants. The task lasted approximately 40 min.

The auditory memory task consisted of five blocks, including 40 trials for each. Trials began with a tone pip. After a silence of 0.5 s, a sequence of three morae was presented to each ear simultaneously. The sequences in left and right ears contained different morae that were randomly chosen from 30 kana characters, i.e., the Japanese syllabary. Each mora was presented only once during a trial, for example, /na/, /ho/, and /ke/ in the left ear and /yu/, /mu/, and /no/ in the right ear. The three morae were presented with a stimulus onset asynchrony of 0.5 s. The partial-report method was conducted in four of five blocks. After one of four possible delay periods (0, 1, 2, or 4 s), participants verbally reported the three morae that they heard in either the left or right ear. For their report, a visual cue (a white circle with a visual angle of 2.0 deg and an eccentricity of 4.0 deg) appeared on either the left or right side of the computer screen with a viewing distance of 57 cm. Full-report procedures were used in the remaining block. Participants reported all six morae presented in left and right ears without a delay. The partial-report blocks comprised 160 trials, whereas the full-report block consisted of 40 trials. The order of the blocks was randomized among participants. This task lasted approximately 1 h.

### Data analyses

**Figure 1:**
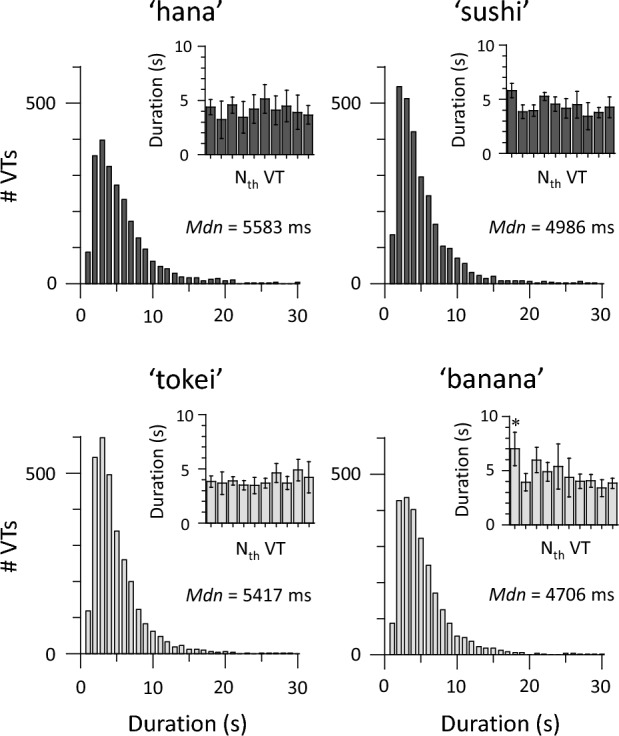
Temporal dynamics of four VT trials (*N* = 45). Stimuli were chosen from 2- and 3-mora words (shown in black and gray, respectively). VTs were pooled from all participants. Histograms of VT durations followed a log-normal distribution. Median VT durations did not differ among the four types of trials. There was no difference in duration between initial and subsequent VTs for any trial, except the ‘banana’ trial. Error bars indicate the standard error of the mean. *p < 0.05.

We obtained verbal form sequences from four types of VT trials. Durations of each verbal form (henceforth, VT durations) were longer than 500 ms. Thus, there was no response derived from inaccurate VTs^23^. In the literature of perceptual multistability^25,26^, the duration to the first transition is longer than to subsequent ones. The first 10 successive VT durations were extracted from each trial (shown in the insets in Fig. 1). There was no difference between first and subsequent VT durations for any trial, except the ‘banana’ trial (paired-sample *t*-tests, *p* < 0.05, Bonferroni correction for multiple comparisons). The first VT durations were excluded from subsequent analyses. We used χ^2^ tests to check whether histograms of VT durations were fitted by a gamma or log-normal distribution. Although VT durations with fewer than 30 s were included in this analysis, more than 99% of all data were covered, regardless of trial type. We used the median of VT durations as a representative value for each participant because VT duration data did not follow a normal distribution. We performed a one-way analysis of variance (ANOVA) to compare VT durations across trials.

We calculated the number of VTs for each participant. Shapiro–Wilk tests showed that data of VT numbers followed a normal distribution: *W* > 0.980, *p* = 0.61. A Smirnov–Grubbs test did not reveal any outliers of VT numbers. We computed Pearson’s correlation coefficients between the number of VTs under 2- and 3-mora conditions. We split each 5-min trial into five 1-min periods to further examine temporal dynamics of VT data. The number of VTs and the proportion of VT durations were calculated for each period. We performed a repeated-measure ANOVA on the number of VTs. Because the proportion of VT durations did not follow a normal distribution, the data were arcsine-transformed and applied to ANOVA. To further examine dynamics of VTs, we computed the transition ratio between possible forms. We counted the number of VTs between each pair of possible forms and divided it by the total number of VTs observed in each trial.

In the auditory memory task, we computed the proportion of correct responses to make partial-report accuracy (max. 3 correct responses) compatible with full-report accuracy (max. 6 correct responses). We estimated Pearson’s correlations when investigating the relationship between auditory memory and VTs. In addition, multivariate permutation tests were used to precisely assess these correlations. We estimated the probability of obtaining the correlation by a random projection of all values for each condition. The probability was determined by permuting values 10,000 times and establishing the proportion of random correlations that was higher than those obtained empirically. Statistical analyses were carried out with IBM SPSS Statistics (version 25) and R (version 3.5.1).

## Results

### Dynamics of VTs

VT durations obtained from all participants were pooled for each trial to check dynamics of VTs (Fig. 1). For all trials, χ^2^ tests showed that histograms of VT durations followed a log-normal distribution (χ^2^ < 17.61, *p* = 0.014), rather than a gamma distribution (χ^2^ < 160.24, *p* < 0.001). This is consistent with previous findings indicating that the distribution of durations is well fitted by a log–normal function in the other perceptual bistability: auditory streaming and visual plaids^26,27^. Median VT durations did not differ between four types of the trials: *F*(3, 132) = 1.92, *p* = 0.13, ${\eta }_{p}^{2}$ = 0.04. Durations (mean ± SD) were 5647 ± 3352 ms for ‘hana’, 5016 ± 2510 ms for ‘sushi’, 4729 ± 2602 ms for ‘tokei’, and 5423 ± 4629 ms for ‘banana.’ The time constant of VTs (around 5 s) was similar to those of auditory streaming (around 8 s) and visual plaids (around 4 s)^26^. Thus, dynamics of VTs were within the range expected from previous findings.

**Figure 2:**
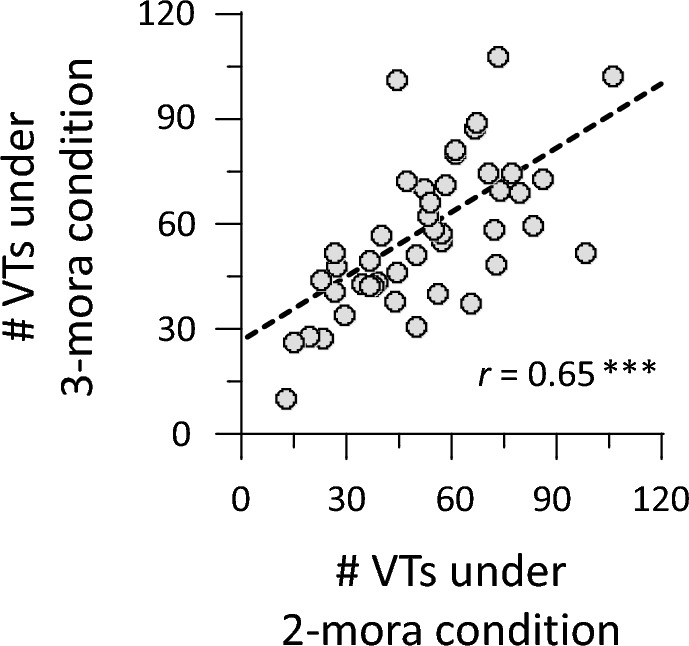
Positive correlation between VT numbers of 2- and 3-mora conditions. The 2-mora conditions consist of ‘hana’ and ‘sushi’ trials, whereas 3-mora conditions contain ‘banana’ and ‘tokei’ trials. Circles indicate individual data (*N* = 45). A dashed line represents a linear regression fit. ***p < 0.001.

To compare results between 2- and 3-mora conditions, we averaged the number of VTs in the ‘hana’ and ‘sushi’ trials and in the ‘tokei’ and ‘banana’ trials. Participants reported 52.6 ± 6.7 occurrences of VTs in 2-mora conditions and 57.1 ± 6.7 occurrences of VTs in 3-mora conditions. VTs tended to occur more frequently in 3-mora than in 2-mora conditions, although the difference was not significant (paired-sample *t*-test, *t*(44) = 1.66, *p* = 0.105, Cohen’s *d* = 0.21). This is consistent with our hypothesis that VTs increase under 3-mora conditions, due to faster adaptation to the current verbal form in auditory memory. We also found significant correlation between 2- and 3-mora conditions: *r* = 0.65, *p* < 0.001 (Fig. 2). This positive correlation indicates the consistency of VTs (irrespective of phonemes) for each individual.

### Temporal changes in VTs

**Figure 3:**
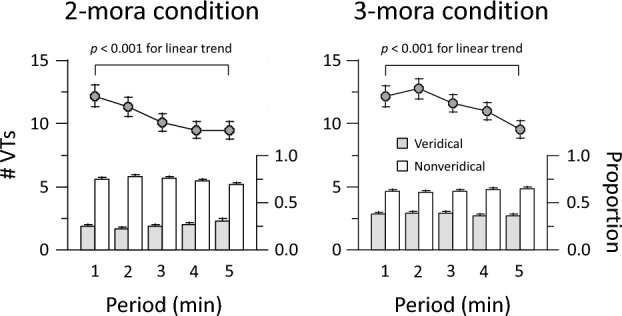
Numbers of VTs (lines) and proportion of verbal forms (bars) for each 1-min period. Veridical forms include ‘hana’ and ‘sushi’ for 2-mora conditions, and ‘banana’ and ‘tokei’ for 3-mora conditions. Nonveridical forms contain three types of possible percepts for each trial (see Methods section for details). Error bars represent the standard error of the mean.

We classified a 5-min trial into five 1-min periods to investigate temporal changes in VTs (Fig. 3). A 2 (mora) × 5 (period) ANOVA showed that the number of VTs did not differ between 2- and 3-mora conditions (10.5 ± 5.0 and 11.4 ± 4.9; *F*(1, 44) = 2.74, *p* = 0.105, ${\eta }_{p}^{2}$ = 0.06). The main effect of period was significant (*F*(4, 176) = 17.39, *p* < 0.001, ${\eta }_{p}^{2}$ = 0.28). There was a linear trend of smaller VT numbers over time for 2-mora conditions (*F*(1, 44) = 15.84, *p* < 0.001, ${\eta }_{p}^{2}$ = 0.27) and for 3-mora conditions (*F*(1, 44) = 18.01, *p* < 0.001, ${\eta }_{p}^{2}$ = 0.29). The interaction between mora and period was not significant (*F*(4, 176) = 2.42, *p* = 0.084, ${\eta }_{p}^{2}$ = 0.05). VTs occurred more frequently in the present study (12.2, 12.1, and 10.9 occurrences) than in a previous study (4.5, 7.6, and 8.5 occurrences for the first, second, and third 1-min periods). In that study, the number of VTs was smaller during an initial 30-s period than during subsequent periods when one-syllable English words, such as ‘tray’ and ‘crash,’ were repeatedly presented^13^. Participants were instructed to report verbal forms orally whenever VTs occurred, but no data were obtained after 3 min. In this study, possible forms were presented beforehand so that participants could easily report their perceptions. Thus, it should be noted that the number of VTs is affected by stimulus properties and task procedures.

Similarly, we performed a 2 × 5 ANOVA on the proportion of verbal forms (Fig. 3). The proportion of veridical forms was greater for 3-mora conditions (0.379 ± 0.082) than for 2-mora conditions (0.251 ± 0.105) (*F*(1, 44) = 67.70, *p* < 0.001, ${\eta }_{p}^{2}$ = 0.61). A main effect of period was not significant (*F*(4, 176) = 1.05, *p* = 0.38, ${\eta }_{p}^{2}$ = 0.02). However, the interaction was significant (*F*(4, 176) = 4.34, *p* = 0.002, ${\eta }_{p}^{2}$ = 0.09). Participants tended to hear veridical forms longer during the first to fourth 1-min periods for 3-mora conditions than for 2-mora conditions. This suggests that participant perception stays on veridical forms for a longer period under 3-mora conditions.

**Figure 4:**
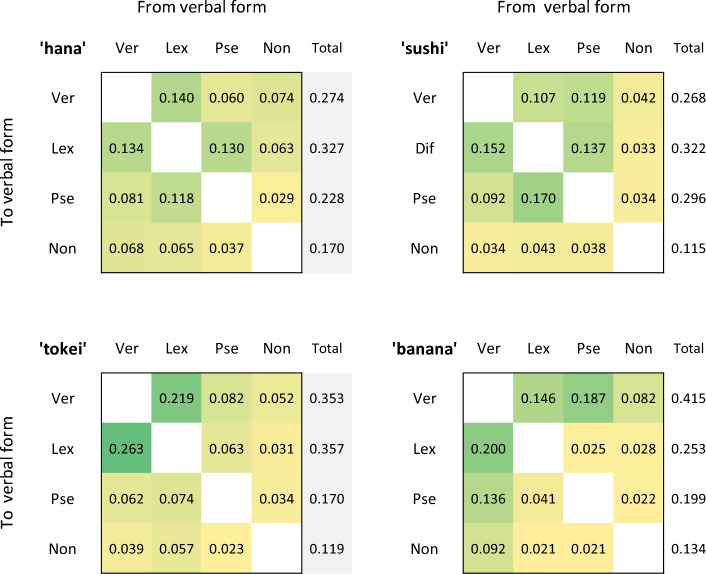
Results of transition ratio between possible forms for each trial. *Lex* lexical different, *Non* nonsense, *Pse* pseudoword, *Ver* veridical.

Figure 4 shows the group mean ratio of transitions between possible forms of the four stimuli. Dark-green and light-yellow shaded cells indicate higher and lower transition ratios, respectively. The transition ratio to veridical forms was higher under 3-mora conditions (0.353 for ‘tokei’ and 0.415 for ‘banana’) than under 2-mora conditions (0.274 for ‘hana’ and 0.268 for ‘sushi’). Perhaps the strength of a word template under 3-mora conditions leads to stabilization of veridical forms. Thus, it is likely that VTs under 2-mora conditions reflect purely sensory adaptation to possible verbal forms.

### Persistence of auditory memory

In addition to the VT task, participants performed an auditory memory task in which two different sequences of three morae were presented to the left and right ears simultaneously. In partial-report blocks, participants recalled one of the two sequences, i.e., from either the left or right ear, after one of four possible delay periods: 0, 1, 2 and 4 s. In full-report blocks, they recalled all six morae immediately after stimulus presentation.

**Figure 5:**
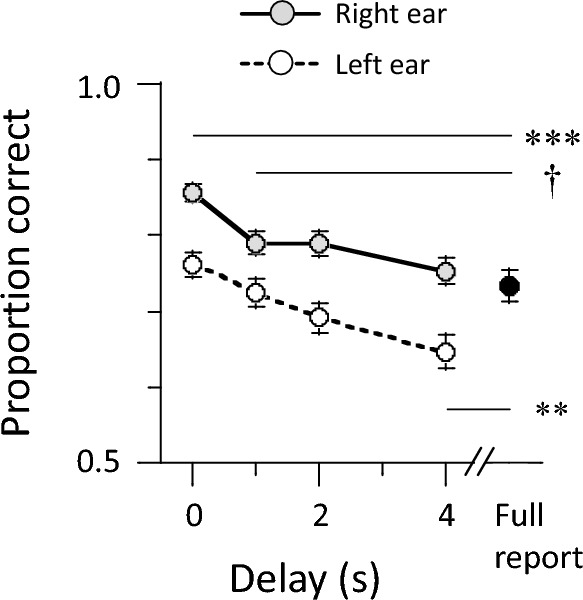
Group mean of free recall accuracy in the auditory memory task (*N* = 45). Gray-shaded and open circles indicate mean accuracy in recalling right- and left-ear sequences, respectively, during partial-report blocks. Each sequence contained three morae. Participants verbally reported targets after one of four possible delay periods (0, 1, 2 and 4 s). Recall accuracy decreased with an increase of delay periods. The black circle represents mean accuracy in full-report blocks where participants had to recall all six morae, i.e., from both ears, immediately after stimulus presentation. Error bars represent the standard error of the mean. ***p < 0.001, **p < 0.01, †*p* < 0.10.

Figure 5 shows the group mean of free recall accuracy in the auditory memory task. Gray-shaded and open circles indicate accuracy in recalling sequences presented to the right and left ears, respectively. We performed a 2 (ear) × 4 (delay) repeated-measure ANOVA on correct responses in the partial-report blocks. On average, participants scored higher when having to recall sequences presented to the right (0.797 ± 0.088) than the left ear (0.707 ± 0.113) (*F*(1, 44) = 36.34, *p* < 0.001, ${\eta }_{p}^{2}$ = 0.45). A right-ear advantage for linguistic stimuli has been interpreted to reflect processes that probably take place in the language-dominant left hemisphere^28,29^, which could explain the present result. In addition, the ANOVA result showed that recall accuracy decreases as the delay periods increase (*F*(3, 132) = 37.19, *p* < 0.001, ${\eta }_{p}^{2}$ = 0.46). There was no interaction between ear and delay (*F*(3, 132) = 2.06, *p* = 0.111, ${\eta }_{p}^{2}$ = 0.05).

Finally, the black circle in Fig. 5 represents the group mean accuracy in the full-report block. We averaged correct responses across trials each delay period per participant, collapsed recall accuracy across the two ears, and submitted partial- and full-report accuracies to a paired-sample *t*-test. Partial-report accuracies with 0-s and 1-s delays (0.808 ± 0.077 and 0.757 ± 0.097) were greater than full-report accuracy (0.735 ± 0.140): *t*(44) = 5.31, *p* < 0.001, Cohen’s *d* = 0.66;* t*(44) = 1.87, *p* = 0.068, Cohen’s *d* = 0.19. However, recall accuracies did not differ between 2-s delay partial reports (0.740 ± 0.110) and full reports (*t*(44) = 0.46, *p* = 0.65, Cohen’s *d* = 0.05). Conversely, partial-report accuracy with 4-s delay (0.700 ± 0.110) was lower than full-report accuracy (*t*(44) = 2.93, *p* = 0.005, Cohen’s *d* = 0.28). Even in the case of immediate recall, participants may forget memory items when orally answering them. Thus, our results indicate that delayed recall accuracy of a few seconds better reflects persistence of auditory memory.

### Coupling between auditory memory and VTs

**Figure 6:**
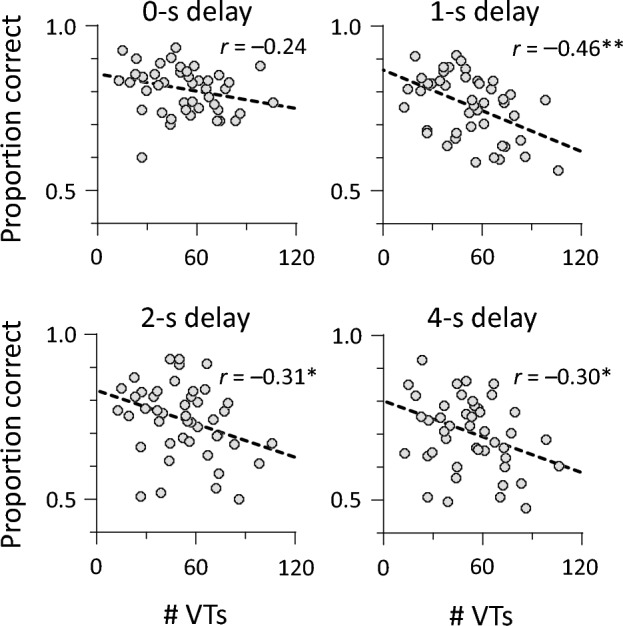
Correlations between VTs and auditory memory under 2-mora conditions. The number of VTs was negatively correlated with the proportion of correct responses for all delay conditions. A dashed line represents a linear regression fit. **p < 0.01. *p < 0.05.

**Figure 7:**
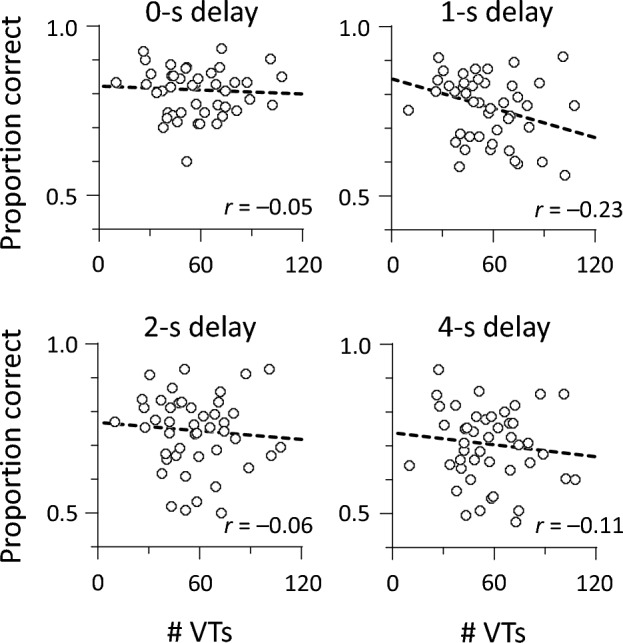
Correlations between VTs and auditory memory under 3-mora conditions. No correlation reached statistical significance (*p*s > 0.125). A dashed line represents a linear regression fit.

Figures 6 and 7 show the total number of VTs (x-axis) in 2- and 3-mora conditions, respectively, and the proportion of correct responses in the auditory memory task (y-axis) scored separately for the four delay periods. Importantly, all conditions showed more or less negative correlations. The results indicate that participants with the longer persistence of auditory memory have fewer VTs, supporting our hypothesis that auditory memory is closely linked with auditory perceptual organization. Specifically, under 2-mora conditions, Pearson’s correlations were significant at 1-, 2-, and 4-s delays (*r* = − 0.46, *p* = 0.002; *r* = − 0.31, *p* = 0.041; *r* = − 0.30, *p* = 0.043), but not at 0-s delay (*r* = − 0.24, *p* = 0.109). We performed multivariate permutation tests for the correlations. A significant correlation for 1-s delay survived (*p* = 0.005), whereas those for 2-s and 4-s delays did not (*p* = 0.085 and *p* = 0.072). Importantly, 1-s memory persistence, rather than 2-s and 4-s memory persistence, better predicted durations of each verbal form. In contrast, no correlation under 3-mora conditions reached statistical significance (|*r*| < 0.23, *p* = 0.125). The number of VTs was not correlated with full-report accuracy (*r* = − 0.24, *p* = 0.113 for 2-mora conditions; *r* = − 0.02, *p* = 0.88 for 3-mora conditions). Thus, memory for immediate recall is not associated with the number of VTs.

The number of VTs decreased over time (Fig. 3). We examined whether the number of VTs in the first or second half of the trial contributes to negative sensory–perceptual correlations mentioned above (Figs. 6 and 7). The total number of VTs was divided into first- and second-half periods (150 s for each) (28.6 ± 12.6 and 24.0 ± 10.6 occurrences in 2-mora conditions; 31.0 ± 12.1 and 26.0 ± 11.2 occurrences in 3-mora conditions). We calculated Pearson’s correlations between VT numbers and auditory memory (Table 1). Under 2-mora conditions, the magnitude of correlation coefficients was greater for the first half (range − 0.50 to − 0.25) than for the second half (range − 0.35 to − 0.17), regardless of the delay period. Under 3-mora conditions, no correlations reached statistical significance (|*r*| < 0.24, *p* = 0.119). Memory persistence was not associated with VTs affected by temporal decay, suggesting that sensory–perceptual correlations are susceptible to other factors.

**Table 1:** Sensory–perceptual correlations (*N* = 45).

# VTs	2-mora condition				3-mora condition			
	1st half		2nd half		1st half		2nd half	
	*r*	*p*	*r*	*p*	*r*	*p*	*r*	*p*
Auditory memory								
0-s delay	− 0.25	0.095	− 0.17	0.25	− 0.01	0.97	− 0.10	0.50
1-s delay	− 0.50	< 0.001	− 0.35	0.018	− 0.20	0.19	− 0.24	0.12
2-s delay	− 0.37	0.016	− 0.19	0.21	0.03	0.82	− 0.14	0.35
4-s delay	− 0.36	0.014	− 0.21	0.16	− 0.08	0.61	− 0.15	0.32

The number of VTs was split into first- and second-half periods (150 s for each). For auditory memory, delayed recall accuracy was computed using partial-report procedures (see the “Methods” section for details).

## Discussion

The present results demonstrated that memory persistence is closely linked to auditory perceptual organization. Specifically, delayed recall accuracy was negatively correlated with the number of VTs under 2-mora conditions. This indicates that individuals with longer memory persistence have longer VT durations, supporting the concept that adaptation to perceptual objects is involved in perceptual multistability. However, there was no significant correlation between delayed recall accuracy and VTs under 3-mora conditions. Thus, sensory–perceptual correlations disappear when stimulus words in VTs depend strongly on veridical forms. We will discuss the relationships in terms of psychological findings, computational models, and neural correlates.

We showed negative correlations between memory persistence and VT numbers. Computational models have argued that dynamics of perceptual multistability are affected by adaptation, inhibition, and noise^14–17^. Specifically, it is postulated that perceptual switching mechanisms are based on mutual inhibition between neural populations coding perceptual objects^15,30,31^. Neuroimaging studies have demonstrated that levels of γ-aminobutyric acid (GABA), i.e., inhibitory neurometabolite, in brain regions are involved in perceptual switching in binocular rivalry, auditory streaming, and VTs^24,32,33^. Strong mutual inhibition between different objects appears to induce slow perceptual switching. On the other hand, neurophysiological studies have shown that formation and selection of auditory objects can be explained by adaptation of neuronal responses in the auditory cortex and brainstem^34,35^. However, the functional substance of sensory adaptation was unknown. Here, we provide a functional account for switching mechanisms. Long persistence of auditory memory leads to slow adaptation to sensory inputs, resulting in a reduced number of VTs.

It is intriguing that memory persistence and VT durations have similar time scales of a few seconds. Specifically, 1-s memory persistence better predicted formation and selection of auditory objects. The current form is selected from possible forms, determined by matching with sensory inputs, and attenuated within this time window. This idea is consistent with predictive coding, which produces robust and stable perception from noisy and ambiguous sensory inputs^36,37^. In this framework, predictions generated in the brain are compared with sensory inputs. Prediction errors are fed back to perceptual systems and predictions are updated to minimize prediction errors. Several researchers have argued that prediction errors are modulated by adaptation or neural fatigue in low-level brain areas^38,39^. A recent study proposed that formation and selection of auditory objects originate from multiple hierarchical levels: peripheral-, central-, and object-analysis stages^18^. In the literature on VTs, the supramarginal gyrus is responsible for temporary storage of phonological information, whereas the inferior frontal cortex generates predictions or prediction errors via reference to gestural knowledge^22,40,41^. Thus, we can imagine that individual differences in VTs occur in the pathway from the auditory area to the frontal area^42,43^.

We did not find significant sensory–perceptual correlations under 3-mora conditions that tended to move participant perception toward veridical forms. Thus, these correlations are probably affected not only by low-level sensory adaptation, but also by top-down processing, such as schema matching^3,10^. In addition, there is a view that although adaptation stabilizes perception, it is not sufficient to induce perceptual switching^14^. Several researchers have argued that perceptual switching is mainly caused by noise, e.g., attentional orienting and eye blinks^31,44,45^. Thus, we speculate that the susceptibility of VTs is associated with the absence of correlations under 3-mora conditions.

The present study has some limitations. First, it is possible that weighting of factors affecting VTs changes over time. Sensory–perceptual correlations differed between the first- and second-half periods. Thus, future studies should clarify whether this is due to temporal decay of memory persistence. Second, perceptual switching can be somewhat, though not completely, modulated by volitional control of participants^26,33^. Participants were given options of verbal forms beforehand and it was easy to indicate their perception. However, there is no way to know whether their attention was focused on a particular verbal form. Finally, VTs are more susceptible to certain personality traits, such as autistic traits, than auditory streaming^46,47^. It is also known that the number of perceptual switching is affected by participant age^48^, although the data in this study were obtained from healthy college students. Therefore, it would be meaningful to compare effect sizes between memory persistence and other factors in future studies.

We found that delayed recall accuracy in the auditory memory task was negatively correlated with the number of VTs, but immediate recall accuracy was not. This indicates that individuals with long-persistent auditory memory adapt slowly to auditory objects. Our results will shed light on perceptual switching mechanisms proposed by computational models. We should consider whether it is possible to generalize sensory–perceptual coupling to perceptual organization.

### Supplementary Information

Supplementary Information.

## Data Availability

Data presented in this study are included in the Supplementary Information, and further inquiries may be directed to the corresponding author.
